# Retrospective Development of an AI Model Combining Ultrasound and Clinical Data for Pediatric Appendicitis Differentiation

**DOI:** 10.1155/emmi/8879232

**Published:** 2025-11-18

**Authors:** Rongying Tan, Yongteng Li, Jianjun Wang, Jixian He, Xiaoting Ding, Yanlin Mou, Xi Zhang, Chen Zhong, Liucheng Yang, Kai Wu

**Affiliations:** ^1^Department of Pediatric Surgery, Zhujiang Hospital, Southern Medical University, Guangzhou 510282, Guangdong, China; ^2^Department of Pediatric Surgery, Zhongshan Boai Hospital, Zhongshan 528403, Guangdong, China

**Keywords:** abdominal ultrasound, artificial intelligence, nonoperative management, pediatric appendicitis, surgical decision support

## Abstract

**Purpose:**

To differentiate complicated appendicitis (CA) from uncomplicated appendicitis (UA) in children, we developed and validated an artificial intelligence (AI) model using a multimodal approach integrating ultrasound images and clinical data.

**Methods:**

A retrospective analysis was performed on 372 pathologically confirmed pediatric appendicitis cases (230 male, 142 female) from three centers, all with preoperative abdominal ultrasound. Deep learning (DL) features and radiomic features were extracted from appendiceal ultrasound images using deep transfer learning (DTL) and conventional radiomic methods, respectively. We selected 12 radiomic features, 9 DL features, and 3 clinical features—namely, white blood cell count (WBC), neutrophil count (NEU), and C-reactive protein (CRP)—for building the machine learning classification model. Based on these features, four distinct models were constructed: the Rad model (radiomic features only), the DL model (DL features only), the DTL model (combined radiomic and DL features), and the Combine model (integrating all three feature types: radiomic, DL, and clinical features). Model performance was evaluated using receiver operating characteristic (ROC) curves, decision curve analysis (DCA), and the DeLong test. Finally, the combined model was compared to the performance of clinicians with varying levels of experience.

**Results:**

The combined model demonstrated consistently favorable performance across all cohorts (AUC: 0.940, 0.895, 0.866, and 0.783 for training and validation sets, respectively). The model's accuracy (0.862) and positive predictive value (0.896) were comparable to senior surgeons (0.741, 0.970) and superior to junior surgeons (0.672, 0.865) in the internal validation cohort. DCA confirmed the clinical utility of the combined model over conventional strategies.

**Conclusion:**

Our ultrasound-based AI model provides reliable differentiation between CA and UA in children, offering potential value as a diagnostic support tool for clinical decision making.

## 1. Introduction

Acute appendicitis is one of the most common causes of abdominal pain in children, with an incidence of 6.7%–8.6% and peak occurrence between 10 and 19 years of age [1–3]. Recent studies have demonstrated that antibiotic therapy can be an effective alternative to surgery for uncomplicated appendicitis (UA), with success rates of up to 89.3% at 1-year follow-up [4]. However, the successful implementation of antibiotic treatment heavily relies on accurate differentiation between UA and complicated appendicitis (CA). CA is primarily treated surgically and increasing numbers of studies have demonstrated favorable results in UA under antibiotic therapy [5–7].

Ultrasonography (US) is considered the optimal imaging modality for diagnosing suspected acute appendicitis in children due to its lack of ionizing radiation and noninvasiveness [3]. While sonographers have demonstrated high diagnostic accuracy for appendicitis detection [8, 9], the systematic classification of CA and UA using ultrasound remains challenging and largely operator dependent. This limitation often leads to diagnostic uncertainty and potentially unnecessary surgical interventions.

Recent advances in artificial intelligence (AI), specifically radiomics and deep learning (DL), have shown promising results in medical image analysis [10, 11]. While previous studies have demonstrated the effectiveness of CT-based imaging characterization in differentiating appendicitis severity in adults [12, 13], few studies have focused on ultrasound-based classification in children. Moreover, existing studies often rely on complex clinical parameters or radiation-exposing imaging modalities [14], which may not be optimal for pediatric patients.

This study aimed to develop and validate an AI model combining DL features, radiomic features, and clinical characteristics from appendiceal US for differentiating CA from UA in children, thereby improving preoperative diagnostic accuracy and providing guidance for clinical decision making.

## 2. Materials and Methods

### 2.1. Patients and Dataset

This retrospective study was conducted in accordance with the Declaration of Helsinki and received approval from the Medical Ethics Committee. We reviewed appendiceal ultrasound images from three centers: Zhujiang Hospital, Southern Medical University (ZJHSMU) (Center 1, January 2014 to December 2023), Zhongshan Boai Hospital (ZSBAH) (Center 2, January 2020 to December 2023), and a public dataset (Center 3) (https://doi.org/10.5281/zenodo.7711412).

**Figure 1:**
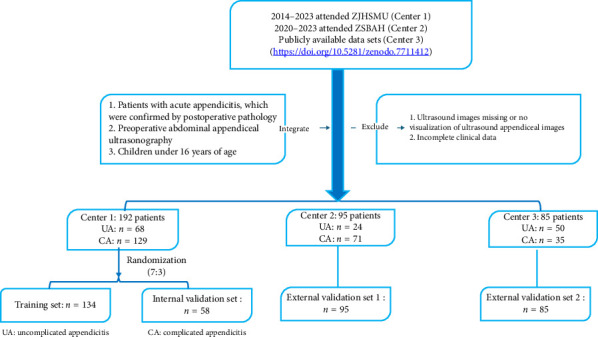
Flowchart of patient enrollment.

Inclusion criteria included the following: ① patients with acute appendicitis, which were confirmed by postoperative pathology; ② preoperative abdominal appendiceal US; and ③ children under 16 years of age. Exclusion criteria included the following: ① ultrasound images missing or no visualization of ultrasound appendiceal images and ② incomplete clinical data. Stratified random sampling of Center 1 children was performed according to the type of postoperative pathology, which was randomly divided into a training set and an internal validation set in the ratio of 7:3. Center 2 and Center 3 served as independent external validation set 1 and validation set 2, respectively. The data in the open dataset were screened using the same inclusion and exclusion criteria.  Figure 1 shows the details of patient screening.

### 2.2. Clinical Characteristics

All the data were obtained from the electronic medical record system, and data entry was performed using a Case Report Form (CRF). The clinical features of the enrolled patients were collected including age, gender, white blood cell (WBC, × 10^9^/L), neutrophil count (NEU, × 10^9^/L), red blood cell (RBC × 10^12^/L), and C-reactive protein (CRP, mg/L). The clinical characteristics were collected when the children were admitted to the hospital, and the blood specimens and US graph were obtained when the children were suspected of having appendicitis (the interval between the two was less than 12 h). Clinical information was collected by two investigators after double checking. To assess the potential for selection bias across the three centers, we compared the baseline characteristics of the study participants.

The gold standard for the typology of acute appendicitis relies on the macroscopic presentation during surgery and the microscopic pathological manifestations. When appendicoliths, perforations, gangrene, and suppurative appendicitis were present, these were classified as CA, while those without these complications were classified as UA.

### 2.3. Region of Interest (ROI) Segmentation and Preprocessing

Ultrasound images (3–10 per patient) were collected at admission by an ultrasonographer (5–10 years' experience). The delineated ROIs were defined to include the entire appendiceal structure, comprising both the lumen and the wall. Specifically, the ROI for each lesion was defined as the smallest rectangular area that encompassed the entire lesion. An experienced radiologist (5 years) performed the segmentation using ITK-SNAP (Version 3.6.0). To ensure segmentation reliability, the ROIs were reviewed by a second radiologist with 10 years of experience.

All selected images underwent standardized preprocessing to reduce the impact of device-specific variations. The pixel values were first normalized to a percentile range of 0.5%–99.5% to effectively minimize outliers. This preprocessing step was essential to address the significant differences in pixel value ranges acquired by different ultrasound devices [15].

### 2.4. Radiomic Features

The handcrafted features can be divided into three groups: (I) geometry, (II) intensity, and (III) texture. The geometry features describe the three-dimensional shape characteristics of the tumor. The intensity features describe the first-order statistical distribution of the voxel intensities within the tumor. The texture features describe the patterns, or the second and high-order spatial distributions of the intensities. Here the texture features are extracted using several different methods, including the gray-level co-occurrence matrix (GLCM), gray-level run length matrix (GLRLM), gray level size zone matrix (GLSZM), and neighborhood gray-tone difference matrix (NGTDM) methods. A total of 1561 handcrafted features were extracted with Pyradiomics software (https://pyradiomics.readthedocs.io) for further statistical analysis.

Feature selection followed a systematic approach: elimination of highly correlated features (Spearman's *ρ* > 0.9) and application of mRMR algorithm to optimize feature relevance. Finally, the least absolute shrinkage and selection operator (LASSO) algorithm was used for feature selection [16]. The optimal penalty parameter (*λ*) was determined via 10-fold cross-validation, based on the minimum criterion. This process forces some regression coefficients to zero, and features with nonzero coefficients were subsequently used for model construction.

### 2.5. DL Features

VGG11, VGG13, VGG16, VGG19, and DenseNet121 as convolutional neural network (CNN) models were pretrained in the ImageNet Large Scale Visual Recognition Challenge-2012 dataset [17]. Before training, the subregion images of the ROI were cropped and were resized to 224 × 224 pixels for the CNN models. To enhance the network's generalization ability and mitigate overfitting in medical image analysis, we employed horizontal flipping and random cropping for data augmentation. Due to limited data, we optimized the learning rate using a cosine decay algorithm (see Supporting Formula S1). Other hyperparameter configurations used in our study were as follows: optimizer—SGD and loss function—SoftMax cross-entropy.

The VGG16 network was selected as the optimal feature extractor based on comparative performance analysis (Supporting Table S1) (area under the ROC curve [AUC] 0.833 in the training set, 0.856 in internal validation). We chose VGG16 as the basic model of our transfer learning model. To mitigate dimensionality effects, DL features (initial dimension: 16,382) were reduced to 64 dimensions via principal component analysis (PCA), thereby enhancing model generalization and reducing overfitting risk. DL features were selected using identical methodology to radiomic feature screening.

**Figure 2:**
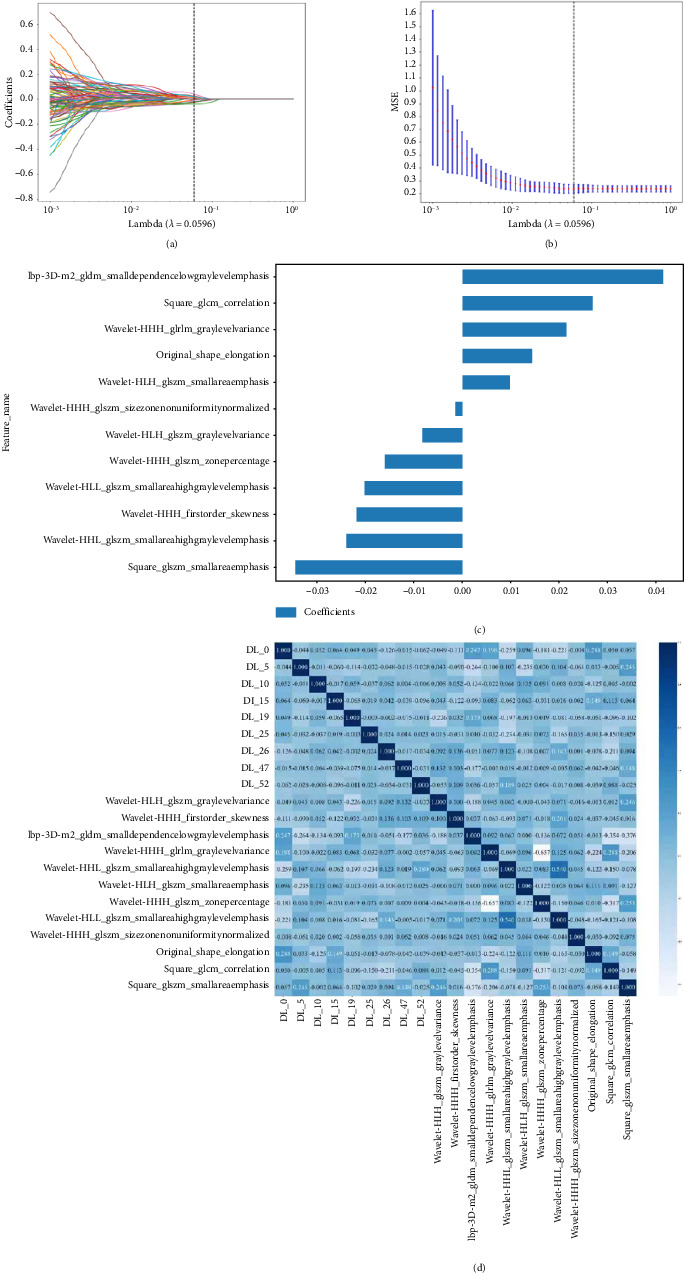
Feature selection analysis. (a) LASSO coefficient profiles of the 105 radiomic features. Vertical line was drawn at the value selected using 10-fold cross-validation, where optimal resulted in 12 nonzero coefficients (*λ* = 0.0596). (b) Regularization parameter tuning identified *λ* = 0.0596 as optimal, minimizing MSE while maintaining model stability. (c) Weight distribution of radiomic features. (d) Spearman correlation between radiomic and DL features.

Following the feature selection process, 12 radiomic features and 9 DL features were identified and subsequently used for model development. Supporting Table S2 summarizes radiomic features, while Figure 2 provides the detailed characteristics.

### 2.6. Construction of Machine Learning Models

**Figure 3:**
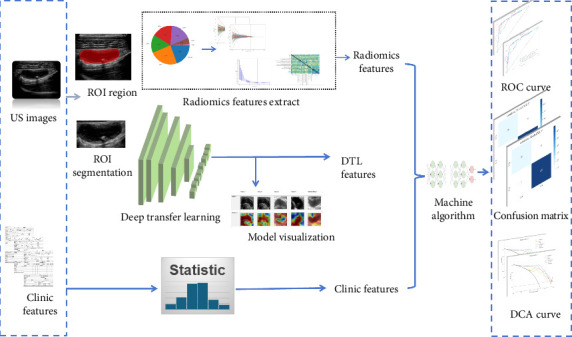
Workflow chart.

Three models were developed: Rad (radiomic features–based), DL (DL features–based), and DLR (combined features). Using the scikit-learn library, we developed and compared several machine learning classifiers—logistic regression (LR), multilayer perceptron (MLP), Light Gradient Boosting Machine (LightGBM), and support vector machine (SVM)—employing 5-fold cross-validation on the training data to optimize parameters and mitigate overfitting. Using the LR method, the Combine model was constructed by combining the DLR signature with the screened clinical features. The DLR model outputs a predictive value, i.e., DLR signature, which is a value between 0 and 1; the higher the value, the more likely it is to be CA.  Figure 3 illustrates the workflow of this study.

### 2.7. Physician Evaluation

Compared with clinical practice, two pediatric surgeons independently evaluated cases from the internal validation and external validation 1 cohorts. External validation 2 (public dataset) was excluded from physician evaluation due to the limited availability of complete clinical information and standardized ultrasound reports and images required for comprehensive physician assessment. The evaluation team included one junior surgeon B (8 years' experience) and one senior surgeon C (15 years' experience). Each surgeon reviewed standardized ultrasound reports along with clinical information (patient symptoms and laboratory tests) while being blinded to the final pathological diagnosis and AI predictions. They classified each case as CA or UA following their routine clinical decision-making process.

### 2.8. Statistical Analyses and Metrics

All statistical analyses were performed using Python (https://www.python.org/). The Shapiro–Wilk test was used to assess the normality of clinical features. Continuous variables were compared using either Student's *t*-test or the Mann–Whitney *U* test, depending on their distribution. Categorical variables were analyzed using chi-squared (*χ*^2^) tests. Statistical significance was defined as a two-sided *p* value ≤ 0.05. Statistically significant characteristics were selected.

To evaluate predictive performance, receiver operating characteristic (ROC) curves were generated for the models. The AUC was calculated as a measure of overall accuracy. The DeLong test was applied to compare the AUC values among the models. Clinical utility was assessed using decision curve analysis (DCA).

## 3. Results

### 3.1. Baseline Clinical Characteristics

A total of 372 children were enrolled in this study, and clinical baseline and univariate analyses for the training set as well as for each validation set are shown in Table 1. The patient cohort consisted of 230 males and 142 females. There were 192 patients in Center 1 (CA: 129; UA: 63), 95 patients in Center 2 (CA 71; UA: 24), and 85 patients in Center 3 (CA 50; UA: 35). The baseline characteristics of patients from the three independent centers are summarized in Supporting Table S3. Statistical comparisons showed that the cohorts were well balanced for key prognostic factors, including age (*p*=0.33) and WBC (*p*=0.077).

Table 1 presents the clinical baseline characteristics across all datasets. Statistical analysis of the training set revealed significant differences between UA and CA groups for WBC, NEU, and CRP values (*p* < 0.05). School-age children (6–12 years) accounted for the majority of subjects (56.6%–79.2%) across all cohorts, consistent with the known peak incidence of appendicitis. Age distributions showed no significant differences between the training and validation sets (all *p* > 0.05), indicating balanced cohort composition. Variables demonstrating statistical significance (WBC, NEU, and CRP) were subsequently incorporated into predictive modeling.

With radiomic features, DL features, and filtered clinic features as inputs, Rad model, DLR model, and Combine model were constructed according to the above feature combination principles.

### 3.2. Selection of the Models

We evaluated the classification performance of the Rad model, DL model, and DLR model under four machine learning classifiers, comparing the training set with each validation set, with the results shown in Table 2. In the DLR model, MLP classifier showed more stable performance across training and validation sets than LR, SVM, and LightGBM, with training AUC/sensitivity/specificity of 0.936 (0.892–0.978)/0.892/0.922 and validation AUCs of 0.732–0.768. The Rad model's SVM classifier exhibited slight overfitting, but steady performance compared to other classifiers, with training AUC/sensitivity/specificity of 0.912 (0.856–0.968)/0.855/0.882. Similarly, for the DL model, SVM demonstrated more stable performance compared to the other three machine learning approaches. Therefore, MLP and SVM were selected as the optimal machine learning algorithms for the DLR model, Rad model, and DL model, respectively (i.e., DLR_MLP, Rad_SVM, and DL_SVM). The combined model (LR) incorporated the DLR-MLP signature together with clinical features (WBC, NEU, CRP). For direct comparison with traditional appendicitis diagnostic tools, this study employed Alvarado and Pediatric Appendicitis Score (PAS) scoring models (both constructed using LR) to differentiate between CA and UA. The AUC (95% CI), sensitivity, and specificity of these models in the training set and each internal validation set are shown in Table 2.

### 3.3. The Performance of the Models

**Figure 4:**
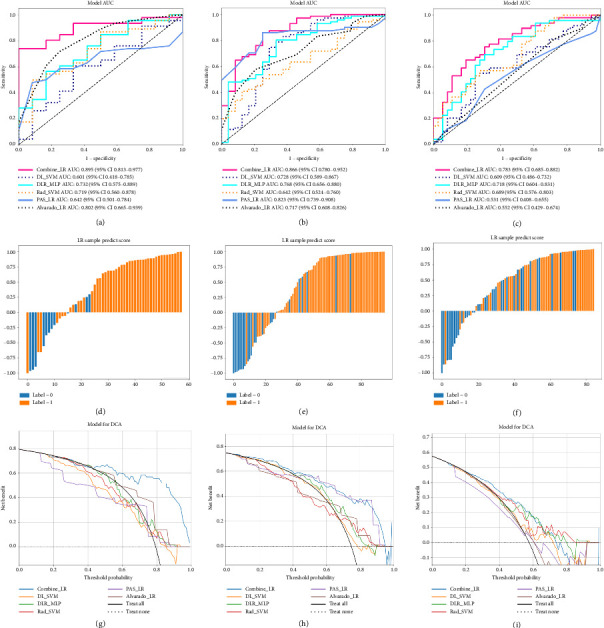
ROC curves of models (Combine, DLR, Rad, and DL), Alvarado score, and PAS score in (a) internal validation, (b) external validation 1, and (c) external validation 2. Prediction histograms for Combine model showing complicated (orange) and uncomplicated (blue) appendicitis in (d) internal validation, (e) external validation 1, and (f) external validation 2. DCA curves for all models in (g) internal validation, (h) external validation 1, and (i) external validation 2.

Among these models, the best-predicted model was the Combine model, whose AUCs (95% CI) for the training set and each validation sets were 0.94 (0.90–0.98), 0.89 (0.81–0.98), 0.87 (0.78–0.95), and 0.78 (0.69–0.88) (Table 2). We also graphically produced ROC curves to show the differentiation of the models (Figures 4(a), 4(b), and 4(c)). The ROC curves demonstrate that the Combine model consistently outperforms other models across all validation datasets, achieving the highest AUC values (0.897–0.929) compared to individual models, particularly showing superior discriminative ability over the Alvarado score and PAS score. The prediction histograms demonstrate effective stratification between CA and UA by the Combine model (Figures 4(d), 4(e), and 4(f)).

Additionally, we performed DeLong tests to assess the statistical significance of differences between the ROC curves of the various models (Table 3). The Combine model consistently outperformed the DL model across all validations (*p*=0.001, 0.026, 0.006) and showed superiority over DLR and Rad in internal and external validation 1. While comparable to the Alvarado score in internal validation, the combined model significantly outperformed the Alvarado score in both external cohorts (*p*=0.023, 0.002). Compared to the PAS, specifically designed for children, the combined model demonstrated statistically significant superior discriminatory ability in both the internal validation and external cohort 2 (*p*=0.001, < 0.001), suggesting its generalizability and applicability for differentiating between CA and UA.

For the evaluation of the clinical effects of each model, we calculated DCA curves for each test set (Figures 4(g), 4(h), and 4(i)). Our DCA revealed that our combined model always provided a better net benefit for predicting CA than the PAS score, Alvarado score, and other models.

### 3.4. Comparison Between the Combine Model and Physicians

Table 4 compares the Combine model performance with physician assessments across validation cohorts. The Combine model demonstrated superior performance to junior physicians with higher AUC (0.895 vs 0.639 in internal validation; 0.866 vs 0.665 in external validation 1), accuracy (86.2% vs 67.2%; 82.1% vs 68.4%), and comparable PPV (89.6% vs 86.5%; 89.7% vs 84.7%). DeLong test results demonstrated that our combined model significantly outperformed junior physicians (*p*=0.005,  0.009) while maintaining diagnostic performance comparable to senior clinicians.

### 3.5. Visualizations of Models

**Figure 5:**
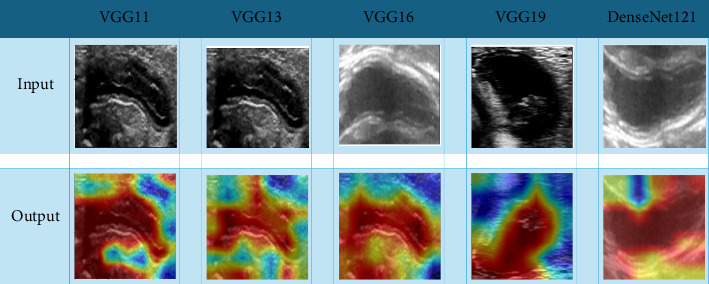
Heatmap of 5 CNN visualizations. The cropped maximum lesion ROI was used as input, and the red area in the output map was the part of the deep learning process where the feature weights were larger.

CNNs were often regarded as “black boxes” [18], and in this study, in order to better understand the feature-weighted ROIs of the CNNs used in making predictions, a gradient-weighted class activation mapping (Grad-CAM) [19] was employed to visualize the visual attention regions of five types of CNNs. Grad-CAM was used to visualize five types of CNNs.  Figure 5 shows the visual attention regions of the CNNs.

## 4. Discussion

Diagnosing acute appendicitis in children remains challenging due to heterogeneous, age-dependent presentations. Particularly in nonverbal infants and young children, nonspecific abdominal signs replace self-reported pain, complicating therapeutic decision making and surgical timing. Previous studies have documented the appendix's immunomodulatory functions and role in sustaining gut microbiota homeostasis [20]. Furthermore, the Malone antegrade continence enema (MACE) procedure—which utilizes the appendix for antegrade irrigation—demonstrates clinical advantages in managing refractory fecal incontinence or severe constipation [21]. Collectively, these findings underscore the appendix's clinical relevance and support its preservation.

We developed and validated the first multimodal model integrating clinical, conventional radiomic, and DL-derived radiomic features for classifying CA in children. The combine model demonstrated significantly superior diagnostic performance (AUC 0.78–0.940) compared to single-modality approaches. Our study has two highlights. First, this study focuses on ultrasound images to differentiate between CA and UA in children. Second, this study uses various CNNs and machine learning methods for feature extraction and modeling. While DL extracts high-level abstract features, radiomics captures quantitative morphological characteristics. This synergistic approach enhances model performance through complementary feature representation.

Unlike previous studies focusing on demographic/clinical features [22, 23], our framework analyzes ultrasound images directly to differentiate CA and UA. Liang et al. and Zhao et al. [12, 13] had shown that imaging characterization using CT images can discern simple appendicitis from nonsimple appendicitis in adults, which demonstrates the feasibility of images in differentiating CA. Marcinkevičs et al. [24] proposed a conceptual bottleneck model (CBM) for the diagnosis, management, and prediction of severity of ultrasound in children with suspicious appendicitis. Compared to Marcinkevičs et al.'s model (AUPR 0.65–0.78) using clinical and ultrasonographic parameters, our radiomics-DL approach achieved superior performance (AUC 0.78–0.94). Kang et al. [25] demonstrated that supplementing a serum biomarker–based binary ML model for appendicitis classification with clinical variables improved AUC from 0.821 to 0.854. Aligning with this observation, our study likewise showed enhanced classification performance following clinical indicator integration into the DLR model (AUC: 0.72–0.73 ⟶ 0.78–0.89).

The clinical laboratory data included in this study are also mentioned in other studies. Güney et al. [14] concluded that the higher the WBC and CRP, the higher the likelihood of CA, and Sengul et al. [26]. showed that NLR can be used as an indicator of the severity of appendicitis in children. The results of the univariate analysis of clinical characteristics in our study suggested that WBC, CRP, and NEU are statistically significant in differentiating CA and UA. Elevated WBC, CRP, and NEU all reflect the degree of inflammatory response of the disease. The clinical indicators included in this study are relatively easy and quick to obtain, even on an outpatient basis through peripheral blood tests. This integration enhances the model's practical utility while maintaining simplicity in clinical implementation. We noted the small sample size in the infant group (< 2 years), reflecting the actual rarity of appendicitis in this age range in clinical practice. Nevertheless, the model's performance in this age group still provides valuable clinical reference.

The combine model's performance was comparable to senior pediatric surgeons (accuracy: 86.2% vs 74.1%) and superior to junior surgeons (67.2%), suggesting its potential value as a diagnostic support tool. Combining clinicians' initial evaluation with AI analysis can expedite risk stratification, facilitating more rapid surgical consultation for high-risk patients (e.g., those with suspected CA) or enabling increased confidence in nonsurgical management of low-risk patients (e.g., those with suspected UA). The DCA demonstrated that our combine model provided greater clinical benefit than conventional approaches in differentiating CA from UA. This is particularly relevant given the growing evidence supporting nonoperative management for UA [2, 5, 6], where accurate preoperative classification is crucial for appropriate treatment selection. The model's high positive predictive value (89.6%) suggests reliable identification of cases requiring immediate surgical intervention. Despite standardized image preprocessing (pixel/size normalization), our model demonstrated reduced performance in external validation cohort 2 (AUC = 0.78, sensitivity = 0.633, and specificity = 0.806). Nevertheless, it maintained superior discriminative ability compared to PAS, Alvarado, and other models. This performance variance likely reflects patient population heterogeneity and domain shift, thereby providing a more realistic assessment of generalizability across diverse clinical environments.

We acknowledge several limitations in our study. First, the retrospective design may introduce selection bias, particularly regarding image quality and case distribution. In our study, exclusion of conservatively managed UA cases—due to reliance on surgical/pathological classification criteria—caused positive–negative case imbalance. Second, although external validation was conducted in two centers, the overall sample size within each cohort was relatively limited, which may constrain the robustness and generalizability of the model. Future studies should employ prospective, multicenter, randomized controlled designs with larger and more diverse populations to enhance external validity. Third, while manual ROI delineation was performed with dual verification, automated segmentation methods could potentially improve reproducibility. Fourth, our study was limited to conventional 2D grayscale images. In clinical practice, appendiceal ultrasound incorporates Doppler flow mapping, dynamic imaging, and sonographic assessment of intra-abdominal fluid/gas—all potentially critical for diagnostic decisions. Future work will implement multichannel learning integrating grayscale and color Doppler images to enhance predictive information and classification robustness.

## 5. Conclusion

In conclusion, our ultrasound-based AI model demonstrates promising performance in differentiating CA from UA in children, with accuracy comparable to experienced pediatric surgeons. This tool could potentially support surgical decision making, particularly in settings where specialist expertise is limited, while adhering to radiation-free imaging principles in pediatric care.

## Figures and Tables

**Table 1:** Characteristic baseline.

	Train		Internal validation		External validation 1		External validation 2	
	CA	UA	CA	UA	CA	UA	CA	UA
Age (*n*/%)								
< 2	0/NA	0/NA	1/8.3	0/NA	0/NA	2/2.8	0/NA	1/3.1
3∼5	3/5.8	7/8.4	0/NA	5/10.87	2/8.3	14/19.7	4/11.1	5/10.2
6∼12	30/58.8	47/56.6	9/75.0	33/71.7	19/79.2	54/76.1	27/75.0	32/65.3
13∼15	18/35.2	29/34.9	2/16.6	8/17.4	3/12.5	1/1.4	5/13.9	11/22.5
Sex (*n*/%)								
F	24/17.9	27/20.1	1/1.7	10/17.2	16/16.8	32/33.7	12/14.1	20/23.5
M	27/20.1	56/41.2	11/19.0	36/62	8/8.4	39/41.0	23/27	30/35.2
WBC	9.89 ± 4.21	16.25 ± 6.69^∗^	10.22 ± 3.51	16.44 ± 5.68^∗^	10.12 ± 4.73	16.11 ± 5.44^∗^	15.06 ± 4.29	17.09 ± 5.78
NEU	7.13 ± 4.34	13.83 ± 6.23^∗^	7.11 ± 3.91	13.57 ± 5.52^∗^	7.55 ± 5.01	14.34 ± 7.45^∗^	12.41 ± 3.78	13.37 ± 4.39
RBC	4.65 ± 0.47	4.73 ± 0.53	5.20 ± 0.27	8.84 ± 0.50	4.66 ± 0.38	4.48 ± 0.53^∗^	4.79 ± 0.30	4.72 ± 0.38
CRP	13.43 ± 19.49	32.89 ± 34.02^∗^	7.42 ± 7.55	55.80 ± 64.94^∗^	15.78 ± 21.14	77.40 ± 83.76^∗^	19.14 ± 25.57	91.75 ± 94.43^∗^

*Note:* Continuous variables: represented as mean ± standard deviation (SD); categorical variables: represented as number. Differences were compared using the *t*-test or Mann–Whitney *U* test. F: female; M: male; NEU: neutrophil count.

Abbreviations: CA, complicated appendicitis; CRP, C-reactive protein; RBC, red blood cell; UA, uncomplicated appendicitis; WBC, white blood cell.

^∗^Indicates *p* < 0.05.

**Table 2:** Performance of models under four machine learning classifiers LR, MLP, LightGBM, and SVM.

		Train			Internal validation			External validation 1			External validation 2		
		AUC (95% CI)	Sen	Spe	AUC (95% CI)	Sen	Spe	AUC (95% CI)	Sen	Spe	AUC (95% CI)	Sen	Spe
Combine		0.94 (0.90–0.98)	0.98	0.82	0.89 (0.81–0.98)	0.72	1.00	0.87 (0.78–0.95)	0.84	0.75	0.78 (0.69–0.88)	0.63	0.81

Alvarado		0.76 (0.68–0.84)	0.27	0.94	0.80 (0.66–0.93)	0.61	0.83	0.72 (0.6–0.83)	0.39	0.91	0.55 (0.42–0.67)	0.51	0.61

PAS		0.73 (0.64–0.82)	0.52	0.76	0.83 (0.56–0.88)	0.54	0.83	0.82 (0.73–0.90)	0.80	0.75	0.53 (0.41–0.65)	0.18	0.80

DLR	LR	0.91 (086–0.96)	0.94	0.78	0.71 (0.55–0.86)	0.61	0.75	0.76 (0.65–0.88)	0.86	0.59	0.68 (0.56–0.80)	0.78	0.53
	SVM	0.97 (0.93–1.00)	0.95	0.92	0.63 (0.46–0.81)	0.48	0.83	0.78 (0.67–0.89)	0.83	0.67	0.70 (0.58–0.81)	0.82	0.58
	LightGBM	0.95 (0.92–0.99)	0.89	0.90	0.67 (0.50–0.85)	0.85	0.42	0.76 (0.63–0.89)	0.79	0.71	0.59 (0.46–0.71)	0.73	0.47
	MLP	0.94 (0.89–0.98)	0.90	0.86	0.73 (0.57–0.89)	0.54	0.83	0.77 (0.66–0.88)	0.79	0.67	0.72 (0.64–0.85)	0.67	0.69

Rad	LR	0.82 (0.74–0.89)	0.64	0.86	0.71 (0.56–0.86)	0.37	1.00	0.60 (0.48–0.72)	0.63	0.63	0.65 (0.52–0.76)	0.63	0.61
	SVM	0.91 (0.86–0.97)	0.86	0.88	0.72 (0.56–0.88)	0.48	0.92	0.64 (0.52–0.76)	0.39	0.88	0.64 (0.55–0.76)	0.55	0.69
	LightGBM	0.93 (0.88–0.97)	0.82	0.94	0.68 (0.53–0.83)	0.61	0.75	0.65 (0.53–0.77)	0.34	0.92	0.54 (0.41–0.66)	0.53	0.61
	MLP	0.87 (0.81–0.93)	0.89	0.71	0.72 (0.55–0.88)	0.78	0.58	0.64 (0.50–0.77)	0.45	0.79	0.69 (0.58–0.80)	0.55	0.72

DL	LR	0.86 (0.80–0.92)	0.78	0.77	0.58 (0.39–0.76)	0.24	0.92	0.77 (0.66–0.89)	0.76	0.75	0.61 (0.49–0.73)	0.59	0.64
	SVM	0.92 (0.86–0.94)	0.87	0.90	0.60 (0.42–0.78)	0.587	0.667	0.73 (0.59–0.87)	0.82	0.62	0.61 (0.48–0.73)	0.51	0.75
	LightGBM	0.92 (0.87–0.97)	0.72	0.92	0.61 (0.43–0.79)	0.478	0.750	0.740 (0.62–0.86)	0.78	0.67	0.58 (0.46–0.71)	0.86	0.31
	MLP	1.00 (1.00–1.00)	0.99	1.00	0.44 (0.23–0.64)	0.696	0.417	0.70 (0.57–0.82)	0.73	0.62	0.55 (0.42–0.67)	0.51	0.67

*Note:* AUC: area under the receiver operating characteristic curve; ACC: accuracy; Sen: sensitivity; Spe: specificity; MLP: multilayer perceptron.

Abbreviations: CI, confidence interval; LightGBM, Light Gradient Boosting Machine; LR, logistic regression; SVM, support vector machine.

**Table 3:** DeLong test comparison among models.

	Internal validation	External validation 1	External validation 2
Combine vs DLR	0.017	0.002	0.087
Combine vs Rad	0.021	< 0.001	0.101
Combine vs DL	0.001	0.026	0.006
Combine vs Alvarado	0.143	0.023	0.002
Combine vs PAS	0.001	0.498	< 0.001

**Table 4:** Comparing the Combine model with the doctors' interpretations.

	Internal validation					External validation 1				
	ACC	AUC (95% CI)	PPV	NPV	*P* ^∗^	ACC	AUC (95% CI)	PPV	NPV	*P* ^∗^
Combine	0.862	0.895 (0.812–0.977)	0.896	0.700	NA	0.821	0.866 (0.781–0.952)	0.897	0.630	NA
Junior	0.672	0.639 (0.479–0.799)	0.865	0.333	0.005	0.684	0.665 (0.552–0.771)	0.847	0.417	0.009
Senior	0.741	0.806 (0.7000.911)	0.970	0.440	0.204	0.832	0.777 (0.673–0.880)	0.887	0.667	0.227

*Note:* ACC: accuracy. *P*^∗^: DeLong test values comparing the combined model with physicians of two different experience levels.

Abbreviations: AUC, area under the curve; CI, confidence interval; NPV, negative predictive value; PPV, positive predictive value.

## Data Availability

The datasets used and/or analyzed during the current study are available from the corresponding author on reasonable request.

## Supporting Information

Supporting Formula S1: the learning rate in our study. Supporting Table S1: five convolutional neural network models were compared in the training set and the internal validation set, and the results. Supporting Table S2: descriptions of the selected radiomic features. Supporting Table S3: baseline characteristics across the three centers.
