# Supplementary material for: Retrospective Development of an AI Model Combining Ultrasound and Clinical Data for Pediatric Appendicitis Differentiation
Source: Emerg Med Int. 2025 Nov 18;2025:8879232. doi: 10.1155/emmi/8879232 (PMC12646731; doi:10.1155/emmi/8879232)
Supplement: Supporting Information — Additional supporting information can be found online in the Supporting Information section. [file 8879232.f1.docx]

**Supplementary Material**

1. **Formula S1**

In our study, the learning rate was defined as follows:

$$\eta_{t}=\eta_{min}^{i}+\frac{1}{2}\left( \eta_{max}^{i}-\eta_{min}^{i} \right)\left( 1+cos\left( \frac{T_{cur}}{T_{i}}\pi\right) \right)$$

The notation$\eta_{\min}^{i}=0$ sets the minimum learning rate, while $\eta_{\max}^{i}=0.01$

establishes the maximum learning rate. The term$T_{i}=60$ denotes the number of epochs in the iterative training process.

2. **Table S1** Five convolutional neural network models were compared in the training set and the internal validation set, and the results.

**Table S1.** Comparison of the performance of 5 convolutional neural networks (CNNs).

| Model Name |  | AUC（95% CI） | ACC | Sen | Specificity | PPV |
| --- | --- | --- | --- | --- | --- | --- |
| vgg11 | train | 0.753（0.671-0.835） | 0.664 | 0.531 | 0.882 | 0.88 |
|  | internal validation | 0.853(0.7433-0.9632) | 0.776 | 0.761 | 0.833 | 0.946 |
| vgg13 | train | 0.676(0.5835-0.7692) | 0.597 | 0.506 | 0.745 | 0.764 |
|  | internal validation | 0.795(0.6514-0.9392) | 0.81 | 0.848 | 0.667 | 0.907 |
| vgg16 | train | 0.833(0.7645-0.9010) | 0.769 | 0.795 | 0.725 | 0.825 |
|  | internal validation | 0.856(0.7257-0.9862) | 0.845 | 0.848 | 0.833 | 0.951 |
| vgg19 | train | 0.677(0.5846-0.7686) | 0.664 | 0.675 | 0.647 | 0.757 |
|  | internal validation | 0.813(0.6751-0.9517) | 0.655 | 0.587 | 0.917 | 0.964 |
| densenet121 | train | 0.853(0.7909-0.9154) | 0.761 | 0.663 | 0.922 | 0.932 |
|  | internal validation | 0.788(0.6466-0.9295) | 0.741 | 0.717 | 0.833 | 0.943 |

AUC: area under the receiver operating characteristic curve; CI: confidence interval; ACC: accuracy; PPV: positive predictive value; Sen: Sensitivity; Spe: Specificity.

The vgg16 was selected as the DL feature extraction neural network. From the avgpool layer output of the trained vgg16 model, after PCA feature dimensionality reduction and lasso feature filtering, a total of 9 DL features were selected.

3. **Table S****2** Descriptions of the selected Radiomics features.

| Feature category | Feature name | Description |
| --- | --- | --- |
| Wavelet Features | wavelet-HLH_glszm_GrayLevelVariance | Measures the variance in gray-level intensities in the GLSZM matrix after wavelet HLH decomposition. |
|  | wavelet-HHH_firstorder_Skewness | Measures the asymmetry of the gray-level intensity distribution after wavelet HHH decomposition. |
|  | wavelet-HHH_glrlm_GrayLevelVariance | Measures the variance in gray-level intensities in the GLRLM matrix after wavelet HHH decomposition. |
|  | wavelet-HHL_glszm_SmallAreaHighGrayLevelEmphasis | Measures the proportion of small-sized zones with high gray-level values after wavelet HHL decomposition. |
|  | wavelet-HLH_glszm_SmallAreaEmphasis | Measures the proportion of small-sized zones in the image after wavelet HLH decomposition. |
|  | wavelet-HHH_glszm_ZonePercentage | Provides the ratio of the number of zones to the total number of voxels in the ROI after wavelet HHH decomposition. |
|  | wavelet-HLL_glszm_SmallAreaHighGrayLevelEmphasis | Measures the proportion of small-sized zones with high gray-level values after wavelet HLL decomposition. |
|  | wavelet-HHH_glszm_SizeZoneNonUniformityNormalized | Measures the variability of zone size volumes throughout the image, normalized by the total number of zones, after wavelet HHH decomposition. |
| Shape Features | original_shape_Elongation | Measures the ratio of the largest principal component to the second largest principal component, reflecting the elongation of the ROI. |
| LBP Features | lbp-3D-m2_gldm_SmallDependenceLowGrayLevelEmphasis | Measures the proportion of small dependence with lower gray-level values using Local Binary Pattern (3D-m2) transformation. |
| Square Transformation Features | square_glcm_Correlation | Measures the linear dependency of gray-level values to their respective voxels in the GLCM matrix after square transformation. |
|  | square_glszm_SmallAreaEmphasis | Measures the proportion of small-sized zones in the image after square transformation. |

4. **Table S3** Baseline Characteristics Across the Three Centers

| **Features** | **ALL** | **Center 1** | **Center 2** | **Center 3** | **p-value** |
| --- | --- | --- | --- | --- | --- |
| Age | 10.40±3.28 | 10.55±3.35 | 9.97±3.06 | 10.54±3.36 | 0.33 |
| WBC | 14.73±5.97 | 14.23±6.42 | 14.60±5.86 | 15.08±4.79 | 0.077 |
| NEU | 12.16±6.24 | 11.57±6.28 | 12.62±7.50 | 12.08±4.17 | 0.155 |
| RBC | 4.73±0.48 | 4.76±0.53 | 4.63±0.46 | 4.76±0.35 | 0.056 |
| CRP | 56.69±65.80 | 51.68±49.03 | 61.80±77.84 | 62.25±82.21 | 0.317 |
| Sex (Man) | 167(44.89%) | 87(45.31%) | 48(50.53%) | 32(37.65%) | 0.219 |

Continuous variables: represented as mean ± standard deviation (SD); Categorical variable: number(n%). Differences were compared using the t-test; Mann–Whitney U test; WBC: white blood cell, NEU: neutrophil count, RBC: red blood cell; CRP:C-reactive protein.
